# Supplementary figures and images for: Unusually low genetic divergence at COI barcode locus between two species of intertidal Thalassaphorura (Collembola: Onychiuridae)
Source: PeerJ. 2018 Jun 18;6:e5021. doi: 10.7717/peerj.5021 (PMC6011825; doi:10.7717/peerj.5021)

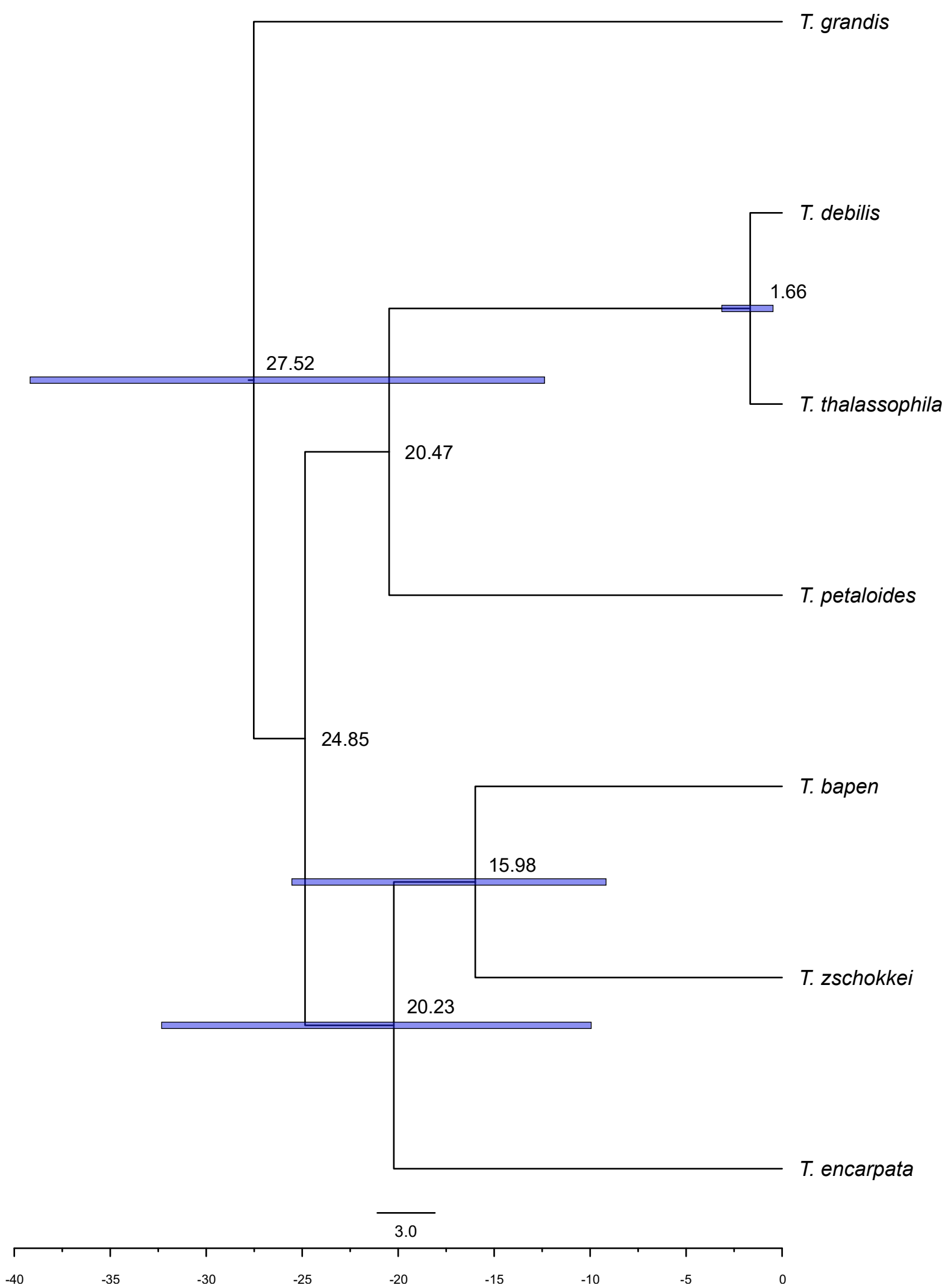

Supplement: Supplemental Information 3 [file peerj-06-5021-s003.pdf]
